# Associations between life’s essential 8 and sarcopenia in US adults: a cross-sectional analysis

**DOI:** 10.1038/s41598-024-59421-9

**Published:** 2024-04-20

**Authors:** Feng Long, Su Zou, Youhai Dong

**Affiliations:** 1 Department of Orthopedics, Shanghai Fifth People’s Hospital, Fudan University, Shanghai, China; 2 Department of Cardiology, Shanghai Fifth People’s Hospital, Fudan University, Shanghai, China

**Keywords:** Cardiology, Diseases

## Abstract

Cardiovascular disease (CVD) is closely associated with sarcopenia. We aimed to examine the relationship between Life’s Essential 8 (LE8) and the incidence of sarcopenia among adults in the United States. In this study, a cross-sectional analysis was conducted using data from the National Health and Nutrition Examination Survey from 2013 to 2018 and included 5999 adult participants. LE8 score was categorized into low (< 49), moderate (49–79), and high CVH (≥ 79) groups and consisted of health behavior score and health factor score based on American Heart Association definitions. Sarcopenia was defined according to The Foundation for the National Institutes of Health Sarcopenia Project. Multivariate logistic regressions, restricted cubic spline regressions, and subgroup analyses were used to assess the association between LE8 and sarcopenia. LE8 and its subscales score were negatively associated with the incidence of sarcopenia in US adults.

## Introduction

Sarcopenia is the age-related loss of skeletal muscle mass and functions as a strong predictor of adverse health outcomes, including a higher risk of falls, fracture^1,2^, physical disability^3^, metabolic impairments^4^ and increased mortality^5^. The prevalence of sarcopenia among people aged 60–70 years old is reported to be 5–13%, but it increases to 11–50% in people older than 80^6^. It is becoming a major public health problem^7^. With increasing age, tissue is confronted with less muscle protein and more fat mass^8^. Furthermore, genetics^9^, nutritional status^10^, physical activity^11^ and selective mortality^12^ are the risk factors for sarcopenia. Additionally, sarcopenia is also associated with respiratory disease^13^, cognitive impairment^14^, and cardiovascular disease^15^. Studies found that sarcopenia is an important risk factor for CVD^16^ and interestingly, CVD, in turn, can accelerate the process of sarcopenia^17^. Increasing evidence demonstrate the close relationship between sarcopenia and CVD.

In contrast to Life’s Simple 7 (LS7) score proposed by the American Heart Association (AHA) in 2010, LE8 score, an updated quantification algorithm in 2022, added sleep health^18^ element and updated definitions and scores for the previous 7 components (diet, physical activity, nicotine exposure, body mass index, blood lipids, blood glucose, and blood pressure)^18^ to quantify cardiovascular health (CVH). In the past decade, there is substantial evidence that LS7 has some imitations on the assessment of healthy lifestyles in modern burdensome social environments and different individuals^18^. Compared with LE7, LE8 is more sensitive to interindividual differences and intraindividual variations^18^.

Given extensive evidence indicating the close association between CVD and sarcopenia, LE8, the assessment tool for quantification of CVH may be a potential evaluation criterion for the risk of sarcopenia. As yet, no study has investigated the relationship between LE8 and sarcopenia. Therefore, this study aimed to assess the association between LE8 and sarcopenia using the available National Health and Nutrition Examination Surveys (NHANES) data. We hypothesized that sarcopenia and LE8 score have a dose–response relationship and participants with sarcopenia have a lower LE8 score than the nonsarcopenic.

## Materials and methods

### Study design and participants

NHANES is a survey designed to examine the health and nutritional status of the population in US involving interviews and physical examinations. The survey has been conducted in 2-year cycles from 1999 to 2000 and 3 cycles conducted from 2013–2014 through 2017–2018 were used in this study. These data were publicly available on the NHANES official website (http://www.cdc.gov/nchs/nhanes.htm) (accessed on September 18, 2023). Each cycle is independent with different individuals recruited and written informed consent was obtained from every participant.

The total combined sample of NHANES 2013–2018 comprised 29,400 participants and 17,057 participants aged 20 years old and above were included. We excluded participants with incomplete information for sarcopenia (n = 10,564), all 8 LE8 metrics (n = 493), and education levels (n = 1). The final study population included 5999 participants.

### Measurement of LE8

The LE8 score includes health behaviors (diet, physical activity (PA), nicotine exposure, and sleep health) and health factors (body mass index (BMI), blood lipids, blood glucose, and blood pressure)^18^. Each component metric is scored on a scale of 0 to 100 and the total LE8 score is calculated as the average of the 8 components. Detailed algorithms of LE8 score have been published and can be found in the Journal of Circulation^18^. Four metrics of health behaviors are collected by self-reported questionnaires and components of health factors are measured with physical examinations. Diet metric was measured by self-reported daily intake and the scores were calculated using data from two 24 h dietary recalls and evaluated by the Healthy Eating Index (HEI) 2015^19^. Physical activity scores were calculated based on the frequency, duration, and intensity of activity per week from self-report questionnaires. Nicotine exposure included inhalational nicotine delivery systems use or secondhand smoke exposure. Sleep health scores were measured by average hours of sleep per night. Additionally, BMI was calculated as the weight in kilograms divided by height in meters squared (kg/m^2^). Blood specimens from participants were used to measure blood parameters, such as blood lipids, fasting blood glucose, and hemoglobin A1C, through high-performance liquid chromatography or enzymatic assay. Blood pressure was measured after resting quietly in a sitting position for 5 min in the mobile examination center (MEC) and the average of three consecutive blood pressure measurements were used for analysis.

### Measurement and definition of sarcopenia

According to guidelines, the diagnosis of sarcopenia depends on the physical examinations. Appendicular lean mass (ALM), the sum of lean mass in the arms and legs, was measured using dual-energy X-ray absorptiometry (DXA). DXA was performed on individuals by Hologic Discovery model A densitometers (Hologic, Bedford, Massachusetts, USA). A value of ALM standardized to BMI (ALM_BMI_) was used to define sarcopenia^20^ based on the criteria designated by a recent consensus meeting known as the “Foundation for the National Institutes of Health Sarcopenia Project”. Men were considered to have sarcopenia if the index ALM_BMI_ < 0.789 and women < 0.512^21^.

### Covariates

The information regarding demographic characteristics and lifestyle components was collected from questionnaires released by NHANES. In this study, age was stratified into two strata: 20–39 years or ≥ 40 years. Race included four categories: Mexican American; non-Hispanic white; non-Hispanic black, and others. Family poverty income ratio (PIR) was categorized as low (PIR < 1.3), medium (PIR = 1.3–3.5), and high (PIR > 3.5). Education level was categorized as Less than 9th grade, 9th to 11th grade, High school graduate, Some college and College graduate or above. Marital status was categorized as married/living with a partner or others and home status was classified as rented, owned/being bought, or others. Smoking status was categorized as never smoked, current smoker, or former smoker. Hypertension was defined as systolic blood pressure ≥ 140 mm Hg or diastolic blood pressure ≥ 90 mm Hg, diagnosed by a doctor, or use of prescription for hypertension. Coronary heart disease was defined as diagnosed by a doctor. Diabetes was defined as glycated hemoglobin HbA1c ≥ 6.5%, fasting glucose ≥ 7 mmol/L, random blood glucose ≥ 11.1 mmol/L, 2-h OGTT blood glucose ≥ 11.1 mmol/L, diagnosed by a doctor, or use of diabetes medication or insulin.

### Statistical analysis

Because of the complex sampling design of NHANES, all data analyses considered sampling design and sampling weight to represent all US population^22^. This study combined the NHANES cycles from 2013–2014 through 2017–2018 and proper weights were used for weighted analysis. Continuous variables were summarized as means and standard deviation and analyzed using the Mann–Whitney U test or the student’s t-test. Categorical variables were presented as frequencies and percentages and compared using the chi-square test. Multivariable logistic regression models were used to determine the OR and 95% CI for the relationship between LE8 scores and sarcopenia. Model 1 was adjusted for age, race, and gender. Model 2 was adjusted for the factors included in model 1 and PIR, education levels, marital status, and home status. Additionally, LE8 score, health behavior score, and health factor score were used as categorical variables in the logistic regression models and a trend test was performed. The restricted cubic spline model was used for the dose–response analysis. Sensitivity analysis was used to test the robustness of the results. We excluded participants who had histories of CVD and perform multivariable logistic regression analysis to investigate the association between LE8 and its health behavior and health factors subscales. Additionally, propensity score matching (PSM) was used to reduce selection bias in observational studies. All statistical analyses were performed using R version 4.1.2 (R Foundation for Statistical Computing, Vienna, Austria) and a two-sided P < 0.05 was considered statistically significant.

### Ethical approval

The data in this study were publicly available on the NHANES website. All procedures involving human participants were approved by the National Center for Health Statistics Research Ethics Review Committee, and all participants signed informed consent forms. This study complied with guidelines for cross-sectional studies outlined in the Strengthening the Reporting of Observational Studies in Epidemiology (STROBE)^23^. NHANES is a survey designed to examine the health and nutritional status of the population in US involving interviews and physical examinations. Each cycle is independent with different individuals recruited and signed informed consent was obtained from every participant.

## Results

### Participants characteristics

**Figure 1:**
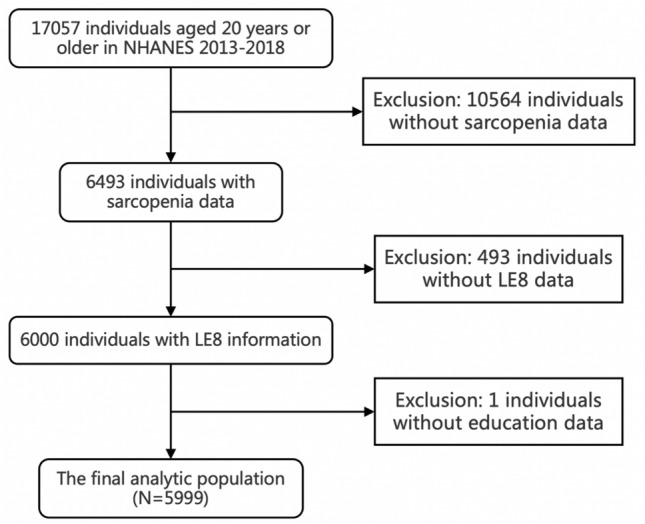
The participant enrollment procedure. Inclusion and exclusion process for the final analysis was based on the 2013–2018 National Health and Nutrition Examination Survey.

The demographic characteristics of 5999 participants were included. A flowchart of the study selection is shown in Fig. 1 and baseline characteristics of participants are presented in Table 1. The weighted mean age of the study population was 39.44 (95% CI 39.18–39.70) years, and 3177 were female (52.96%) and participants with sarcopenia were significantly older than the nonsarcopenic (mean 39.19 vs 42.69 y; P < 0.0001). The mean LE8 score was 71.25 and the percentages of low, moderate, and high CVH were 8.45%, 62.96%, and 28.59% separately. LE8 score, HEI 2015 total score, health behavior score and health factor score were significantly lower of people with sarcopenia than the non-sarcopenia. For the LE8 elements, diet score had the least mean score of 37.31, whereas blood glucose score had the highest mean score of 90.04. Except for nicotine exposure score and sleep health score, there were statistically significant differences with lower means across all the 8 LE8 components’ scores for sarcopenia status. Besides, participants with sarcopenia had lower PIR and education levels, and higher values of age, and BMI, and incidence of hypertension, coronary heart disease, and diabetes compared to those without sarcopenia.

**Table 1:** Characteristics of participants in the NHANES 2013–2018 cycles.

Variable	Participants			
	Total (n = 5999)	Without sarcopenia (n = 5474, 91.25%)	With sarcopenia (n = 525, 8.75%)	*P* value
Age, years	39.44 ± 0.26	39.19 ± 0.28	42.69 ± 0.66	< 0.0001
Age strata				< 0.001
20–39	2974(49.57)	2789(50.84)	185(39.46)	
≥ 40	3025(50.43)	2685(49.16)	340(60.54)	
PIR strata				< 0.0001
< 1.3	1742(29.04)	1548(21.36)	194(31.65)	
1.3–3.5	2321(38.69)	2106(34.96)	215(42.70)	
≥ 3.5	1936(32.27)	1820(43.69)	116(25.65)	
BMI	28.69 ± 0.15	28.17 ± 0.13	35.54 ± 0.44	< 0.0001
Gender				0.51
Male	2822(47.04)	2570(48.69)	252(51.01)	
Female	3177(52.96)	2904(51.31)	273(48.99)	
Race				< 0.0001
Non-Hispanic White	2151(35.86)	2009(61.16)	142(45.78)	
Non-Hispanic Black	1178(19.64)	1148(11.66)	30(3.55)	
Mexican American	929(15.49)	743(9.47)	186(26.80)	
Other Hispanic	650(10.84)	572(7.17)	78(12.20)	
Other race	1091(18.19)	1002(10.54)	89(11.66)	
Education levels				< 0.0001
Less than 9th grade	304(5.07)	223(2.46)	81(11.10)	
9–11th grade	670(11.17)	598(7.88)	72(10.58)	
High school graduate	1314(21.9)	1171(21.94)	143(31.48)	
Some college	1991(33.19)	1846(33.45)	145(33.01)	
College graduate or above	1720(28.67)	1636(34.28)	84(13.83)	
Marital status				0.97
Married/living with a partner	3680(61.34)	3338(61.80)	342(61.91)	
Others	2319(38.66)	2136(38.20)	183(38.09)	
Home status				0.01
Rented	2516(41.94)	2271(35.67)	245(44.89)	
Owned/being bought	3235(53.93)	2977(60.48)	258(51.16)	
Others	248(4.13)	226(3.85)	22(3.95)	
Smoking status				0.91
Never	3714(61.91)	3377(60.49)	337(61.16)	
Current	1281(21.35)	1185(20.20)	96(19.45)	
Former	1004(16.74)	912(19.30)	92(19.39)	
Hypertension	1691(28.19)	1476(25.31)	215(43.32)	< 0.0001
Coronary heart disease	62(1.03)	47(0.83)	15(3.54)	< 0.001
Diabetes	570(9.5)	459(6.46)	111(19.78)	< 0.0001
CVH				< 0.0001
Low (< 49)	507(8.45)	414(6.33)	93(19.27)	
Moderate (49–79)	3777(62.96)	3397(60.29)	380(72.47)	
High (≥ 79)	1715(28.59)	1663(33.39)	52(8.26)	
LE8	71.25 ± 0.38	72.06 ± 0.38	60.61 ± 0.75	< 0.0001
Diet score	37.31 ± 0.93	37.84 ± 0.97	30.28 ± 1.97	0.001
Physical activity score	79.21 ± 0.76	80.28 ± 0.66	64.98 ± 3.30	< 0.0001
Nicotine exposure score	71.81 ± 0.94	71.66 ± 0.96	73.81 ± 1.56	0.17
Sleep health score	83.94 ± 0.55	84.14 ± 0.57	81.32 ± 1.51	0.08
Body mass index score	61.76 ± 0.80	64.01 ± 0.73	31.86 ± 1.79	< 0.0001
Blood lipid score	68.75 ± 0.67	69.35 ± 0.72	60.87 ± 1.68	< 0.0001
Blood glucose score	90.04 ± 0.46	91.04 ± 0.43	76.76 ± 1.63	< 0.0001
Blood pressure score	77.20 ± 0.57	78.13 ± 0.59	64.96 ± 1.62	< 0.0001
HEI 2015 total score	50.11 ± 0.40	50.36 ± 0.41	46.78 ± 0.83	< 0.001
Health behaviors score	68.07 ± 0.49	68.48 ± 0.52	62.60 ± 1.03	< 0.0001
Health factors score	74.44 ± 0.39	75.63 ± 0.38	58.62 ± 0.90	< 0.0001

Data are presented as unweighted number for categorical variables and mean (SE) for continuous variables.

*LE8* life’s essential 8, *HEI* healthy eating index, *CVH* cardiovascular health, *HEI* healthy eating index.

### Association between LE8 score and sarcopenia

**Figure 2:**
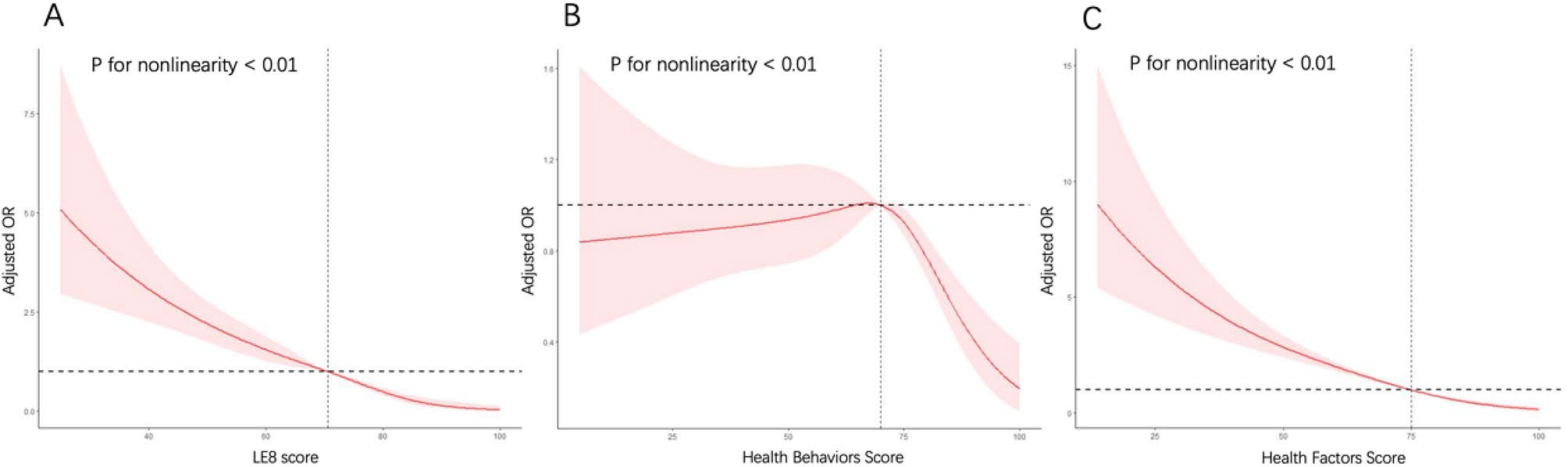
Dose–response relationship between LE8 score (**A**), health behavior score (**B**), health factors score (**C**), and sarcopenia. Association between LE8 score (**A**), health behaviors score (**B**), health factors score (**C**), and sarcopenia. ORs (solid lines) and 95% confidence levels (shaded areas) were adjusted for age, race and gender, PIR, education levels, marital status, and home status. *LE8* life’s essential 8, *OR* odds ratio,* CI* confidence levels.

The ORs (95% CIs) for the associations between LE8 and the risk of sarcopenia are displayed in Table 2. Compared with low CVH, there was a lower prevalence of sarcopenia in people with high CVH (OR, 0.08; 95% CI 0.05–0.13; P < 0.0001) and moderate CVH (OR, 0.39; 95% CI 0.28–0.55; P < 0.0001). After adjusting for age, race and gender, PIR, education levels, marital status, and home status, participants with high CVH (OR, 0.12; 95% CI 0.07–0.23; P < 0.0001) and moderate CVH (OR, 0.47; 95% CI 0.31–0.72; P < 0.001) still had a lower risk of sarcopenia than low CVH as before (P for trend < 0.0001). The adjusted OR of per 10 scores increase in LE8 score was 0.62 (95% CI 0.56–0.67; P < 0.0001). Accordingly, the association between LE8 and sarcopenia was nonlinear (P < 0.01) in the restricted cubic spline model in Fig. 2A. The minimal threshold for the beneficial association was 70.625 scores (estimate OR = 1).

**Table 2:** Association of the life’s essential 8 scores with sarcopenia.

	Univariable model		Multivariable model 1		Multivariable model 2	
	OR (95% CI)	*p* value	OR (95% CI)	*p* value	OR (95% CI)	*p* value
LE8 score						
Low (0–49)	Ref		Ref		Ref	
Moderate (50–79)	0.39 (0.28, 0.55)	< 0.0001	0.40 (0.28, 0.59)	< 0.0001	0.47 (0.31, 0.72)	< 0.001
High (80–100)	0.08 (0.05, 0.13)	< 0.0001	0.09 (0.05, 0.15)	< 0.0001	0.12 (0.07, 0.23)	< 0.0001
Per 10 points increase	0.58 (0.54, 0.62)	< 0.0001	0.58 (0.54, 0.63)	< 0.0001	0.62 (0.56, 0.67)	< 0.0001
p for trend		< 0.0001		< 0.0001		< 0.0001
Health behaviors score						
Low (0–49)	Ref		Ref		Ref	
Moderate (50–79)	0.99 (0.71, 1.38)	0.97	0.92 (0.65, 1.32)	0.66	1.13 (0.76, 1.69)	0.53
High (80–100)	0.46 (0.29, 0.70)	< 0.001	0.41 (0.26, 0.64)	< 0.001	0.64 (0.39, 1.03)	0.06
Per 10 points increase	0.86 (0.81, 0.91)	< 0.0001	0.84 (0.79, 0.90)	< 0.0001	0.92 (0.85, 0.98)	0.02
p for trend		< 0.0001		< 0.0001		0.033
Health factors score						
Low (0–49)	Ref		Ref		Ref	
Moderate (50–79)	0.35 (0.25, 0.50)	< 0.0001	0.36 (0.24, 0.54)	< 0.0001	0.37 (0.25, 0.57)	< 0.0001
High (80–100)	0.07 (0.05, 0.10)	< 0.0001	0.08 (0.05, 0.12)	< 0.0001	0.08 (0.05, 0.13)	< 0.0001
Per 10 points increase	0.63 (0.60, 0.67)	< 0.0001	0.63 (0.58, 0.68)	< 0.0001	0.64 (0.59, 0.69)	< 0.0001
p for trend		< 0.0001		< 0.0001		< 0.0001

### Association between health behavior score and sarcopenia

Despite the not statistically significant differences in health behavior score strata (P > 0.05), especially after adjusting for the potential covariates, the incidence of sarcopenia in participants with high health behavior score was lower than low health behavior group. After adjusting for age, race and gender, PIR, education levels, marital status, and home status, participants with high (OR, 0.64; 95% CI 0.39–1.03; P = 0.06) and moderate health behavior score group (OR, 1.13; 95% CI 0.76–1.69; P = 0.53) still had a lower risk of sarcopenia than low CVH as before (P for trend = 0.033). In the multivariable regression analysis, the adjusted OR of per 10 scores increase in health behavior score was 0.92 (95% CI 0.85–0.98; P = 0.02). The association between health behavior score and sarcopenia was nonlinear (P < 0.01) in the restricted cubic spline model in Fig. 2B. The minimal threshold for the beneficial association was 70 scores (estimate OR = 1).

### Association between health factor score and sarcopenia

The prevalence of sarcopenia was significantly different among the three strata of health behaviors. After adjusting for age, race and gender, PIR, education levels, marital status, and home status, participants with high (OR, 0.08; 95% CI 0.05–0.13; P < 0.0001) and moderate health behavior score group (OR, 0.37; 95% CI 0.25–0.57; P < 0.0001) still had a lower risk of sarcopenia than low CVH as before (P for trend < 0.0001). In the multivariable regression analysis, the adjusted OR of per 10 scores increase in health factor score was 0.64 (95% CI 0.59–0.69; P < 0.0001). The association between health factor score and sarcopenia was nonlinear (P < 0.01) in the restricted cubic spline model in Fig. 2C. The minimal threshold for the beneficial association was 75 scores (estimate OR = 1).

### Subgroup and sensitivity analysis

**Figure 3:**
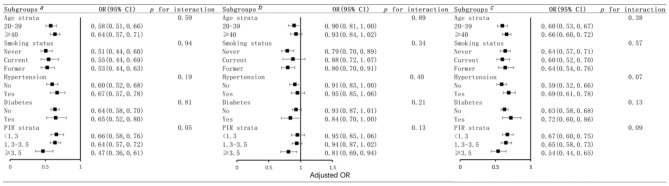
Subgroup analyses between LE8 score (superscript a), health behavior score (superscript b), and health factor score (superscript c) with sarcopenia. Association between LE8 score (superscript a) and its health behavior (superscript b) and health factor (superscript c) subscales with sarcopenia. The stratifications were adjusted for age, race, gender, PIR, education levels, marital status, and home status. ORs were calculated as per 10 scores increase in LE8 score, health behavior score, and health factor score.

Figure 3 shows the result of subgroup analysis. As is shown, after adjusting for age, race, gender, PIR, education level, marital status, and home status, age, smoking status, hypertension, diabetes and, PIR had not a significant interaction effect on the association between LE8 scores and its subscales scores and risk of sarcopenia. LE8 score, health behavior score, and health factor score were significantly negatively associated with sarcopenia in every group. We perform two sensitivity analyses to assess the association between LE8 and its health behavior and health factor subscales with sarcopenia. Considering the potential impact of CVD on LE8 metrics, we investigated the relationship using participants without CVD histories (including coronary heart disease, angina, heart attack, and stroke; n = 217). After excluding participants who had histories of CVD, the OR for per 10 score increase in LE8 score, health behavior score, and health factor score were 0.61 (95% CI 0.55–0.68), 0.91 (95% CI 0.84–0.98) and 0.64 (95% CI 0.59–0.69) separately. In addition, to reduce bias in case selection and clinical confounding factors, we perform propensity score matching to correct the confounding factors (age, race, gender, PIR, education levels, marital status, and home status) and remove bias due to all observed covariates between the sarcopenia and non-sarcopenia and the OR for per 10 score increase in LE8 score, health behavior score and health factor score were 0.59 (95% CI 0.51–0.69), 0.90 (95% CI 0.81–1.00) and 0.62 (95% CI 0.55–0.70), respectively. The results were still robust in sensitivity analyses (Table 3).

**Table 3:** Sensitivity analysis of the association of the life’s essential 8 scores with sarcopenia.

	Excluding CVD history participants^†^		Propensity score matching*	
	OR (95% CI)	*p* value	OR (95% CI)	*p* value
LE8 score				
Low (0–49)	Ref		Ref	
Moderate (50–79)	0.44 (0.29, 0.69)	< 0.001	0.46 (0.28, 0.76)	0.004
High (80–100)	0.11 (0.06, 0.23)	< 0.0001	0.13 (0.07, 0.27)	< 0.0001
Per 10 points increase	0.61 (0.55, 0.68)	< 0.0001	0.59 (0.51, 0.69)	< 0.0001
p for trend		< 0.0001		< 0.0001
Health behaviors score				
Low (0–49)	Ref		Ref	
Moderate (50–79)	1.04 (0.68, 1.60)	0.84	1.36 (0.91, 2.04)	0.13
High (80–100)	0.58 (0.35, 0.98)	0.04	0.60 (0.33, 1.07)	0.08
Per 10 points increase	0.91 (0.84, 0.98)	0.02	0.90 (0.81, 1.00)	0.04
p for trend		0.023		0.054
Health factors score				
Low (0–49)	Ref		Ref	
Moderate (50–79)	0.40 (0.25, 0.63)	< 0.001	0.28 (0.16, 0.47)	< 0.0001
High (80–100)	0.09 (0.05, 0.15)	< 0.0001	0.06 (0.03, 0.12)	< 0.0001
Per 10 points increase	0.64 (0.59, 0.69)	< 0.0001	0.62 (0.55, 0.70)	< 0.0001
p for trend		< 0.0001		< 0.0001

*OR* odds ratio, *CI* confidence interval, *LE8* life's essential 8, *CVD* cardiovascular disease.

^†^Adjusted for age (as continuous variable), race, gender, PIR, education levels, marital status, and home status.

*Matching for age (as continuous variable), race, gender, PIR, education levels, marital status, and home status.

## Discussion

This cross-sectional study found an inverse relationship between LE8 score and sarcopenia. The association was generally robust in sensitivity and subgroup analyses. In contrast to previous studies, this study analyzed data from the NHANES using multivariable regression analysis to assess the relationship between LE8 and sarcopenia in the US adult population. In addition, we addressed the dose–response analysis to reveal an inverse association between the LE8 score, health behavior score, and health factor score with sarcopenia in US adults. To our knowledge, no other studies have examined this specific relationship. The findings from this study suggested that a higher LE8 score was associated with a lower risk of sarcopenia.

Previous studies have reported that cardiovascular disease was associated with sarcopenia. A British prospective cohort study involving 4252 subjects showed that sarcopenia was associated with greater cardiovascular mortality and all-cause mortality^24^. A cross-sectional study with small sample sizes (n = 208) in Brazil reported that sarcopenia was associated with subclinical atherosclerosis and endothelial dysfunction^25^. And in a German cohort, sarcopenia was a frequent co-morbidity among patients with chronic heart failure^26^.

Although the mechanism between CVD and sarcopenia remains not fully understood, current evidence has proved that sarcopenia is significantly associated with the LE8 metrics^27–29^. Sarcopenia and CVD have a common pathogenesis and interaction. In terms of health behavior score, sleep health metric is an influence factor among the four metrics. A meta-analysis indicated that versus the reference category of sleep duration (6–8 h), highest category (more than 8 h) and lowest category of sleep duration (under 6 h) shared the same negative effect regarding increased risk of sarcopenia^30^, almost consistent with sleep health score for CVH. Besides, growing studies about developing effective approaches to counteract the effects of sarcopenia have demonstrated that physical activity has been well accepted to be a significantly effective strategy shown to alleviate sarcopenia^31–33^. According to previous literature, the relationship between physical activity and sarcopenia may be related to inflammation. Inflammation is a well-known factor contributing to sarcopenia progression^34–36^. In exercised muscle, inflammatory cytokines such as IL-6 recruit immune cells and produce IL-10 receptor antagonists decreasing inflammatory injury^37^. Meanwhile, physical activity retards the development of atherosclerosis^38^. Smoking is a well-established risk factor for CVD and several studies have identified smoking is also a risk factor for sarcopenia^39,40^ through impairing muscle protein^41^. Nutrition is generally accepted as an influencing factor of sarcopenia^42,43^.

In the matter of health factor score, obesity, a major risk factor for cardiovascular disease, is closely related to BMI and blood lipids (2 metrics of health factor score) which involves extensive adipose tissue that damages muscle homeostasis, resulting in muscle atrophy and regeneration capacity reduction^44^. Resistin has been considered a proinflammatory molecule that correlates with CVD, expressed by monocytes and macrophages infiltrating the adipose tissue^45^. In skeletal muscle, activation of Insulin receptor substrate-1 and -2 phosphorylation and Akt and AMP-activated protein kinase were impaired by resistin^46^. Adipose tissue has a negative effect on muscle. Diabetes is characterized by hyperglycemia which may lead to loss of skeletal muscle mass and function because of insulin resistance^47,48^. The results of insulin resistance lead to a decrease in protein synthesis, muscle glucose disposal, and an increase in protein degradation^49,50^. As for hypertension, a 5-year follow-up study performed by the National Center for Geriatrics and Gerontology Study of Geriatric Syndromes (NCGG–SGS) found that hypertension was associated with an increased mortality risk among people with sarcopenia^51^. There was a lower cytochrome c oxidase (COX) content in the old hypertensive muscle than in normotensive aging which may lead to rarefaction in the aged skeletal muscle capillary network^52^. Above all, it is a composite score of all the LE8 metrics that relate to sarcopenia and LE8 is a potential tool for the assessment of sarcopenia.

In our investigation, the dose–response relationships showed that LE8 score and health factor score significantly decreased in ORs associated with sarcopenia within the lower range of values and gradually stabilized within the higher range of values. However, the trend was different in the association between health behavior score and sarcopenia. In the low range of health behavior score (value < 70), health behavior score in ORs associated with sarcopenia nearly remain unchanged with the lower range of values and decreased drastically in the higher range of values. A saturation effect was observed in the association between health factor score and sarcopenia, while it was not observed in health behavior score, indicating that stricter health behavior standards may be more desirable. There was no interaction among groups (p for interaction ≥ 0.05) in subgroup analyses which indicated the negative association between LE8 score and sarcopenia was stable in different groups.

There are some potential limitations in this study. First, the current study only included the US population and additional research is required to confirm whether the findings can be generalized to the non-US population. Second, because of the limitations of the NHANES database, our data on health behavior metrics were from self-reported questionary which may be subject to recall bias. Third, residual confounding effects could not be excluded due to measurement errors and unmeasured variables. Fourth, the definition of sarcopenia has different criteria and we used Foundation for the National Institutes of Health (FNIH) criteria which lack functional parameters such as grip strength or gait speed. Finally, as a cross-sectional analysis, the causal relationship between LE8 score and sarcopenia could not be determined in the study. Future longitudinal studies or more sophisticated analytical methods are required to advance our knowledge of this causality.

Despite the limitations mentioned above, this study had several strengths. First, to the best of our knowledge, this is the first study to explore the association between LE8 and sarcopenia. Second, our data using a study sample from the NHANES was relatively large, reliable, and, nationwide. Third, strict inclusion and exclusion criteria were performed and we used propensity score matching and dose–response analysis to measure the stability of results and identified the minimal threshold for the beneficial association.

## Conclusion

In our study, we established an inverse relationship between LE8 and sarcopenia in an adult American population. Our findings suggest that LE8 is a potentially beneficial evaluation criterion for the risk of sarcopenia.

## Data Availability

The data in this study were publicly available on the NHANES official website (http://www.cdc.gov/nchs/nhanes.htm).
